# Is there any benefit to particles over photon radiotherapy?

**DOI:** 10.3332/ecancer.2019.982

**Published:** 2019-12-09

**Authors:** Maria E Goossens, Marc Van den Bulcke, Thierry Gevaert, Lydie Meheus, Dirk Verellen, Jean-Marc Cosset, Guy Storme

**Affiliations:** 1 Cancer Centre, Sciensano (Scientific Institute of Public Health), 1050 Brussels, Belgium; 2 Department of Radiotherapy, University Hospital Brussels, Vrije Universiteit Brussel, 1050 Brussel, Belgium; 3 The Anticancer Fund, Reliable Cancer Therapies, Strombeek-Bever, 1853, Belgium; 4 Iridium Kankernetwerk Antwerp, Belgium; 5 Faculty of Medicine and Pharmacy, Vrije Universiteit Brussel, 1050 Brussel, Belgium; 6 Centre de Radiothérapie Charlebourg, Groupe Amethyst, 65, Avenue Foch, 92250 La Garenne-Colombes, France

**Keywords:** hadron, particles radiotherapy, proton, carbon ion beam, cancer

## Abstract

Particle, essentially, proton radiotherapy (RT) could provide some benefits over photon RT, especially in reducing the side effects of RT. We performed a systematic review to identify the performed randomised clinical trials (RCTs) and ongoing RCTs comparing particle RT with photon therapy. So far, there are no results available from phase 3 RCTs comparing particle RT with photon therapy. Furthermore, the results on side effects comparing proton and carbon ion beam RT with photon RT do vary. The introduction of new techniques in photon RT, such as image-guided RT (IGRT), intensity-modulated RT (IMRT), volumetric arc therapy (VMAT) and stereotactic body RT (SBRT) was already effective in reducing side effects. At present, the lack of evidence limits the indications for proton and carbon ion beam RTs and makes the particle RT still experimental.

## Introduction

Radiotherapy (RT) after surgery is the second main treatment in solid tumours and far in front of systemic treatment [1]. The use of modern RT with image-guided RT (IGRT), intensity-modulated RT (IMRT), volumetric arc therapy (VMAT) and stereotactic body RT (SBRT) drastically decreased the side effects [2, 3]. In the continuous process for improvement, it was suggested that proton therapy, due to its Bragg peak, and carbon ions, due to the Bragg peak and to a higher radiobiologic effect (RBE), could have a promising future and become the best practice in radiotherapy. Randomised clinical trials (RCTs) are the gold standard to compare the effectiveness of one therapy over another [4]. Observational studies can only provide some indications about possible advantages from one therapy compared to another. The recent health technological assessment (HTA) report in Belgium [5] could not show any advantage for proton therapy over photon therapy. The Ludwig Boltzmann Institute published a recent systematic review for carbon ion beam RT [6]. They concluded that ‘carbon ion beam RT (CIRT) can be described as a potentially less invasive cancer treatment due to its physical properties. Due to the lack of controlled trials, no conclusions may be drawn on the comparative effectiveness of CIRT when compared to conventional photon therapy. As of today, CIRT must be considered as experimental treatment’ [6]. We reviewed the literature to identify the results from more recent RCTs on particle RT or ongoing trials.

## Method

Because of the high quality and the extensiveness of both the Belgian HTA report [5] and the systematic review of the Ludwig Boltzmann Institute [6], we chose to update these two reports. The Belgian HTA report closed its search in July 2018, the review on CIRT was closed in August 2017. Therefore, we reviewed the literature for systematic reviews and primary studies comparing particle RT with photon therapy in the databases Medline, EMBASE and Cochrane Library since the closing search dates. Single-arm studies are not included in this study. For CIRT, we searched between September 2017 and February 2019; for proton therapy, we searched between August 2018 and February 2019.

## Results

**Figure 1.**
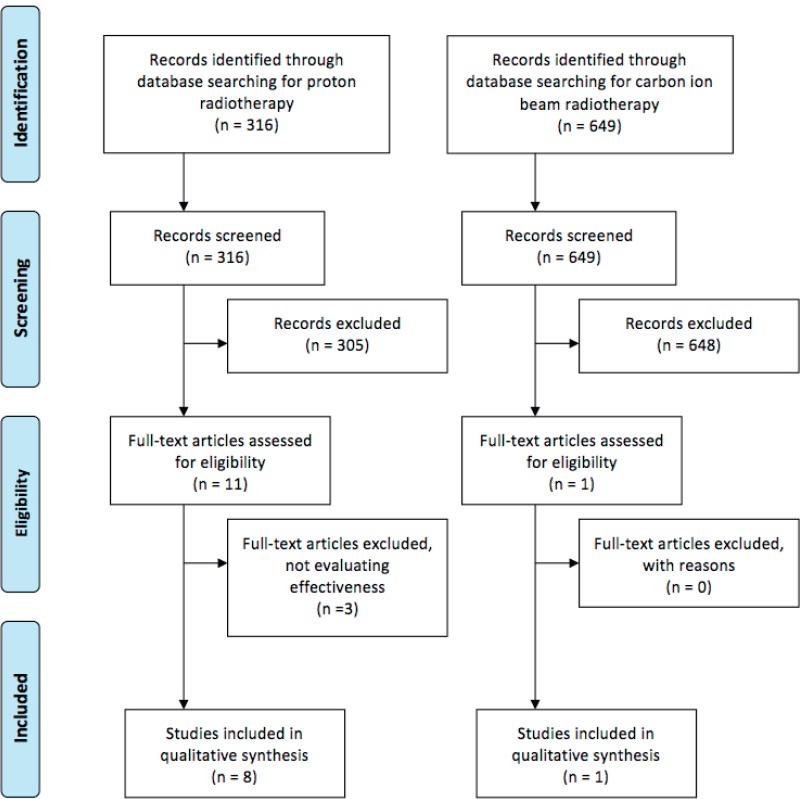
Flow diagram of the published literature stratified for proton and CIRT.

We could identify some new observational studies comparing a photon with particle therapy (Figure 1). The overall survival (OS) in *breast cancer* patients was assessed using the US National Cancer Database between 2004 and 2014. The OS of patients that received proton therapy was not statistically significantly longer than those that received photon therapy [Hazard ratio (HR) 0.85 (95% Confidence interval (CI) 0.68–1.07)] [7].

The preliminary results of a study in *non-small cell lung cancer* with underlying idiopathic pulmonary fibrosis showed a tendency of non-statistically significant better survival compared to X-ray (*p* = 0.08) for patients treated with proton therapy, especially in subgroups of GAP stages II and III at Samsung Medical Center in Korea [8]. In unresectable *hepatocellular carcinoma*, proton RT was associated with improved survival, which may be driven by decreased incidence of post-treatment liver decompensation [Adjusted hazard ratio (AHR) = 0.47 (95%CI 0.27–0.82)] [9].

Proton therapy in patients with *chordomas* and* chondrosarcomas* was associated with improved OS at 5 years, respectively, 100% versus 34.1% (*p* = 0.03) and 75.0% versus 19.1% (*p* = 0.05) using the US National Cancer Database for the years 2003–2014 [10]. A meta-analysis showed that particle therapy was more effective following surgery for chordoma than conventional RT with higher percentages of survival after 10 years for proton therapy [60% (95% CI, 43%–77%)] and CIRT [45% (95% CI, 36%–55%)] [11].

Proton therapy is associated with improved OS [HR 0.47 (95%CI 0.38–0.58)] compared to photon RT for patients with *primary gliomas* in the US National Cancer Database [12].

Consecutive patients with *oesophageal cancer* of the University of Texas MD Anderson Cancer Center receiving proton beam therapy (PBT) were compared with patients receiving intensity-modulated radiation therapy (IMRT). IMRT compared to proton therapy was associated with significantly worse OS [HR 1.45 (95%CI 1.09–1.94)] and worse progression-free survival [HR 1.56 (95%CI 1.19–2.05)] [13]. Shiraishi *et al* [14] performed a propensity matched-based study on key clinical variables in the same institution and found that PBT is associated with significant risk reduction in grade 4 lymphopenia during neoadjuvant chemoradiation therapy in oesophageal cancer.

A recent systematic review for *intracranial benign tumours* considers proton therapy as safe [15].

The overview of recently published included non-randomised comparative studies is presented in Table 1.

There are still no results available from RCTs. A review of clinicaltrial.gov in 2018 did not show any phase 3 RCT for carbon ion radiation therapy [16]. On the other side, there are several initiatives at a European level promoting research on particle therapy. The European particle therapy network (EPTN) creates a firm basis for evidence-based particle therapy at the European level. To achieve this, a work package will set up a worldwide unique prospective data registration programme for nine different tumour sites. Such a programme will provide more insights into the current practice across all European particle therapy centres and into the results of particle therapy with regard to radiation-induced toxicity and efficacy in terms of local control and survival [17, 18]. The European network for light ion hadron therapy (ENLIGHT) is another initiative related to hadron therapy (HT), and focuses on patient selection, clinical trials, technology, radiobiology, imaging and health economics [19]. Another European project summarises the data on carbon ion therapy [20]. Different evidence-based clinical trial strategies can be applied to investigate whether the use of protons over photons is justified: the choice of trial design depends on several factors, such as the primary study objective (efficacy versus prevention), the availability of high quality multivariable normal tissue complication probability (NTCP)-models, financial resources and national reimbursement policies [21].

We could identify seven ongoing phase 3 clinical trials on clinicaltrial.gov (Table 2):

- Radiation therapy with protons or photons in treating patients with liver cancer (ClinicalTrials.gov identifier: NCT03186898)
- Trial of proton versus carbon ion radiation therapy in patients with low and intermediate grade chondrosarcoma of the skull base (CSP12C) (ClinicalTrials.gov identifier: NCT01182753)
- Trial of proton versus carbon ion radiation therapy in patients with chordoma of the skull base (HIT-1) (ClinicalTrials.gov identifier: NCT01182779)
- Comparing proton therapy to photon radiation therapy for oesophageal cancer (ClinicalTrials.gov identifier: NCT03801876)
- Comparing photon therapy to proton therapy to treat patients with lung cancer (ClinicalTrials.gov identifier: NCT01993810)
- Randomised carbon ions versus standard RT for radioresistant tumours (https://clinicaltrials.gov/show/nct02838602, 2016 added to CENTRAL: 31 May, 2018 | 2018 Issue 5 NCT02838602)
- Randomised trial of intensity-modulated PBT (IMPT) versus intensity-modulated photon therapy (IMRT) for the treatment of oropharyngeal cancer of the head and neck (NCT01893307) [22].

## Discussion

While results for particle therapy are still limited to the results of observational studies, there are no results of randomised studies and only a handful of ongoing clinical trials. Most of the trials are superiority trials while the German trial comparing carbon ion RT with proton RT in chondrosarcoma has a non-inferiority design. The lack of results of RCTs is somewhat surprising if we take into account that the first accelerator using protons built primarily for medical use, the Crocker Medical Cyclotron, was completed at Berkeley in 1939 and the first patient was treated in 1954. Regardless of this long track record, today, there is still no clear answer on a possible benefit considering that with modern photon-based approaches such as IGRT, IMRT including VMAT and SBRT dose distributions can be obtained, which challenge the possibilities offered by proton treatment with excellent tumour control and minimal toxicity [3]. Nearly, all newly found retrospective observational studies have reported on OS. Data on toxicity and adverse effect are less likely available in large cancer registries and could only be found in the patient’s electronic health record (EHR). Even there, these data are not always structured and so not available for research. Linking structured data of the EHR with existing cancer registries can bridge a gap between daily practice and research [23, 24]. Furthermore, retrospective observational studies suffer from time-related biases [4, 25, 26]. Immortal time bias can be a major issue and is induced in time-fixed cohort analyses which misclassify unexposed time as exposed time [25, 26]. To overcome these biases, randomised trials will almost always be necessary to show whether the hoped-for benefit of a medical intervention exists [4]. Finally, the technological advancement of the photon beam (IMRT, IGRT, SBRT and VMAT) may also influence OS. Depending on the time period comparing proton with photon RT when these newer techniques were not yet available, OS could be better for proton RT. Finally, also the quality of the facility could influence the OS [27].

The RBE of protons is dependent on dose and on the dose fractionation scheme used. A variety of normal tissue and tumour endpoints has been employed to obtain data on RBE in *in vivo* studies, and it is currently accepted that the RBE value for protons is about 1.1. The major challenge is the inhomogeneity of the tissues and mobility in anatomy causing large variations in tissue density leading to uncertainties in the range of the Bragg peak. These issues need to be addressed in dose calculations and treatment planning, particularly for single field treatments or for distal edges in or close to a critical structure [28]. Recent developments focus on robust treatment planning to compensate for both random (unpredictable) and systematic variables that might influence the dose deposition accuracy [29]. The advantages of proton treatment get lost for the fact that particle treatment is unforgiving for these uncertainties, whereas photons by nature are more robust and forgiving for these issues in real-life clinical situations. Nevertheless, even if protons reduce the low-dose bath, the conformity of the high-dose region immediately adjacent to the target is superior for IMRT [30]. Moreover, we must keep in mind that the neutron-scattered dose is much higher with ‘passive’ (scattering) proton technology than with ‘active’ (pencil beam scanning) proton techniques (PBS-PT) [31]. This PBS PT, particularly intensity-modulated PT, represents the latest advanced PT technology for treating cancers, including thoracic malignancies. However, implementing PBS-PT for moving targets has several additional technical challenges compared with intensity modulated photon radiation therapy or passive scattering PT. Four-dimensional computed tomography-based motion management and robust optimisation and evaluation are crucial for minimising uncertainties associated with beam range and organ motion. Active motion management (e.g. breath-hold), beam gating, rescanning, tracking or adaptive planning may be needed for cases involving significant motion or changes in motion or anatomy over the course of treatment. [32]. Current risk models used with carefully obtained dose distributions predict a second cancer risk reduction for active protons versus photons, but a more or less constant risk of passive protons versus photons [31], while the potential risks of second cancers from scattered proton RT for childhood cancers may cause concern [33]. On the other hand, a recent prospective randomised comparative trial found no differences between intensity-modulated photon therapy (IMRT) and passive scattering proton RT in patients with non-small cell lung cancer [34], and the dose response (the slope of linear ^18^F-FDG-uptake) did not differ significantly between the two modalities [35].

Standard indications for proton RT are melanoma of the eye and uveal tract, brain tumours, certain head and neck tumours and tumours of the base of the skull and of the spine [36, 37]. The benefit could be explained by using the former imaging localisation and fixation of those locations [36, 38]. With exception of the aforementioned pathologies, which in many cases can be challenged with new developments in photon treatment [e.g. high-precision, high doses per fraction treatments such as stereotactic radiosurgery (SRS) and SBRT], there are no other clear indications that favour proton treatment, today. A recent study [39] reviewed the literature for malignancies in children. This review includes also the results from the Belgian HTA report on Hadron RT for children [40] and concluded that while results from phase 3 RCTs are not yet available in paediatric malignancies, clinical outcomes for PT should be favourable with an improved quality of life (QoL), organ function and development and with a reduction in the risk of second malignant neoplasms. On the other hand, ototoxicity was not reduced in children with medulloblastoma [41]. Based on limited data, PBT provides favourable QoL and patient-reported outcome (PRO) profiles for the select brain, head/neck, lung and paediatric cancers [42].

We observe that for proton centres to be financially viable, in addition to the ‘commonly accepted indications’ also ‘conditions of possible benefit’ such as tumours of prostate and lung are considered for treatment [43]. While the ‘commonly accepted indications’ decrease over the years (18.7% in 2006 to 10.6% in 2009), the ‘conditions of possible benefit’ increase from 80% to 89.4% during the same years [43]. As such, the cost of cancer care increases without proof of a real benefit. This is also the case for systemic cancer treatment, which is close to 50% of the financial cost with an improvement in outcome (survival) between 2.5% and 10.0% [44]. A recent study calculated that the overall incremental cost-effectiveness ratio (ICER) for skull base chordoma was €8,855.76/QALY [45].

In The Netherlands, the proton project has a model-based approach based on NTCP for selecting patients, which should be effective as well in outcome as for reducing side effects [46]. After a long period of societal discussion, proton therapy became available in The Netherlands in 2018. This therapy was introduced to The Netherlands in a unique manner. The proton centres have been given permission to treat a maximum of 2,200 patients per year, 4.4% of the total number of patients who receive RT [47]. In Belgium, end-2019, it will be possible for patients to have particle RT at the university hospital of Leuven. Approximately, 100–200 patients a year will be eligible for proton therapy. This number could increase in the future if clinical–scientific trials can determine new indications for proton therapy [48]. Proton therapy will be a reality in Norway from 2023 [15].

Finally, while the level of evidence is still low for proton therapy, the information found on the websites of the proton therapy centres is not always in line with the accepted guidelines and consensus opinion [49].

The technical, radiobiological and financial problems are still more important and complicated with carbon ions. As stated above, no results of any RCT are available today, and ongoing trials comparing carbon ions with protons are limited. The radiobiology of carbon ions is still more complex, with a higher RBE, which varies by a large amount along the Bragg peak. Moreover, the increase in RBE with depth in the stopping region of the particles (the ‘tail’), already mentioned for protons, may pose still more serious complications with carbon ions. Last but not least, the radioprotection problems raised by the use of carbon ions led the International Commission on Radiological Protection (ICRP) to release its publication 127 in 2014 [50]. Finally, the cost of carbon ion facilities exceeds by large the one of a proton centre, and by several orders of magnitude the cost of a modern RT centre [51].

## Conclusion

So far, there are no results available from phase 3 RCTs, neither on side effects nor on outcome comparing particle RT with photon therapy. The introduction of new techniques in photon RT such as IGRT, IMRT, VMAT and SBRT has already proven to be effective in reducing side effects. The lack of evidence limits the indications for proton RT and makes proton RT still experimental. While waiting for the results of the ongoing randomised trials including trials comparing proton RT with IMRT, proton RT should use the best available technique (PSB) and be strictly reserved for selected patients. For carbon ions, the present lack of evidence limits the indications and make this therapy still experimental.

## Conflicts of interest

The authors declare that they have no conflicts of interest.

## Funding statement

None to declare.

## Figures and Tables

**Table 1.** Overview of the included comparative studies.

Author	Data source, country	Indication	N	Comparison	Reported outcome	Results (95%CI)
Chowdhary *et al* [7]	National Cancer Database, USA	Breast cancer	871 723,621	Proton RT Photon RT	Overall survival	0.85 (0.68–1.07)
Kim *et al* [8]	Samsung Medical Center, South Korea	Non-small cell lung cancer	8 22	Proton RT Photon RT	1-Year survival	50 versus 26.4% (*p* = 0.08)
Sanford *et al* [9]	Massachusetts General Hospital, USA	Hepatocellular carcinoma	49 84	Proton RT Photon RT	Overall survival	0.47 (0.27–0.82)
Palm *et al* [10]	National Cancer Database, USA	Chordoma	183 532	Proton RT Photon RT	Overall survival	0.11 (0.01–0.82)
		Chondrosarcoma	54 809	Proton RT Photon RT	Overall survival	0.13 (0.02–0.96)
Zhou *et al* [11]	Meta-analyse, China	Chordoma		Photon RT stereotactic RT proton RT carbon ion RT	10-Year survival	0.21 (0.10–0.33) 0.40 (0.30–0.55) 0.60 (0.43–0.77) 0.45 (0.36–0.55)
Jhaveri *et al* [12]	National Cancer Database, USA	Primary gliomas	170 49,405	Proton RT Photon RT	Overall survival	0.66 (0.53–0.83)
Xi *et al* [13]	MD Anderson Cancer Center, USA	Oesophageal cancer	211 132	IMRT Proton RT	Overall survival	1.45 (1.09–1.94)
			211 132	IMRT Proton RT	Progression free interval	1.56 (1.19–2.05)
Shiraishi et al* [14]	MD Anderson Cancer Center, USA	Oesophageal cancer	136 136	IMRT Proton RT	Grade 4 lymphopenia	0.29 (0.16–0.52)

* propensity-matched study, IMRT: intensity-modulated radiation therapy

**Table 2.** Ongoing phase 3 clinical trials on ClinicalTrials.gov on particle radiotherapy.

ClinicalTrials.gov Identifier	Cancer	Intervention	Comparator	Country	Start date Estimated end date	Primary endpoint
NCT03186898	Liver	Proton RT	Photon RT	USA	June 2017 August 2022	OS
NCT01182753	Chondro-sarcoma	Carbon ion RT	Proton RT*	Germany	August 2010 August 2022	5-Year LPFS
NCT01182779	Chordoma	Carbon ion RT	Proton RT*	Germany	August 2010 August 2023	8-Year LPFS
NCT03801876	Oesophage	Proton RT	IMRT	USA	March 2019 February 2027	OS
NCT01993810	Lung	Proton RT	Photon RT	USA	February 2014 December 2020	OS
NCT02838602	Radio-resistant	Carbon ion RT	Proton RT* IMRT	France	December 2017 November 2023	5-Year PFS
NCT01893307	Oro-pharynx	IMPT	IMRT	USA	August 2013 August 2023	Toxicity

RT, radiotherapy; IMRT, Intensity-Modulated Photon Therapy; IMPT, Intensity-Modulated Proton Beam Therapy; OS, overall survival; LPFS, Local-Progression Free Survival; PFS, Progression-Free Survival

* reference comparator is proton NOT photon radiotherapy
